# Motivations for Restrictive Dieting and Cognitive Restraint Among Polish University Students

**DOI:** 10.3390/nu18162625

**Published:** 2026-08-11

**Authors:** Mateusz Grajek, Alicja Kozik, Tomasz Jurys, Mateusz Rozmiarek

**Affiliations:** 1 Department of Public Health, Faculty of Public Health in Bytom, Medical University of Silesia in Katowice, 41-902 Bytom, Poland; s89995@365.sum.edu.pl; 2 Department of Rehabilitation, Faculty of Health Sciences in Katowice, Medical University of Silesia in Katowice, 40-751 Katowice, Poland; tomasz.jurys@sum.edu.pl; 3 Department of Sports Tourism, Faculty of Physical Culture Sciences, Poznan University of Physical Education, 61-871 Poznan, Poland; rozmiarek@awf.poznan.pl

**Keywords:** motivation, eating disorders, restrictive dieting, risk, cognitive restraint, body dissatisfaction, young adults, students, TFEQ-13, Poland

## Abstract

Background: Restrictive dieting is a common behavior among young adults and may be associated with increased vulnerability to disordered eating. University students represent a population potentially exposed to multiple influences affecting eating behaviors, including academic stress, social pressures, and appearance-related standards promoted through digital media. This study aimed to analyze the relationship between restrictive dietary practices, eating behaviors, and indicators of vulnerability to disordered eating among university students in Poland. Materials and Methods: A cross-sectional study was conducted among 420 university students aged 19–34 years. Data were collected using an anonymous online questionnaire consisting of sociodemographic questions, the original Restrictive Diet Motivation Questionnaire (RDMQ), and the validated Three-Factor Eating Questionnaire-13 (TFEQ-13). Motivational factors were categorized into aesthetic, social, health-related, eating control, and self-discipline, and personal beliefs regarding restrictive dieting. Statistical analyses included the Gamma correlation coefficient, chi-square test, and ordinal logistic regression, with statistical significance set at *p* < 0.05. Results: Restrictive dieting was reported by a substantial proportion of participants, with women reporting this behavior more frequently than men. The most prominent motivations were related to appearance, weight control, social influences, and personal beliefs regarding restrictive diets. Participants who followed restrictive diets demonstrated higher levels of cognitive restraint compared with those who did not engage in restrictive dieting. Strong associations were observed between cognitive restraint and aesthetic motivations, personal beliefs regarding restrictive dieting, social motivations, eating control and self-discipline, and health-related motivations. Multivariable analysis confirmed that aesthetic motivation, personal beliefs, previous restrictive dieting, and female sex were independent predictors of higher cognitive restraint. Conclusions: Restrictive dieting among university students is strongly associated with appearance-related motives, dieting-related beliefs, and increased cognitive restraint. In this study, eating disorder risk was inferred from behavioral indicators, particularly cognitive restraint, rather than from clinical diagnostic criteria. These findings highlight the need for preventive strategies promoting evidence-based nutrition knowledge, healthy eating behaviors, and a positive body image among young adults.

## 1. Introduction

Eating disorders (EDs) represent a significant public health concern, encompassing a broad spectrum of disorders associated with abnormal eating behaviors, body image disturbances, and emotional regulation difficulties. They may occur at any stage of life; however, they most commonly develop during adolescence and young adulthood, making university students a particularly vulnerable population [1,2]. The period of university education is associated with numerous life changes, including increased responsibility, academic pressure, the need for independent functioning, relocation to a new place of residence, and uncertainty regarding future career prospects. These factors may contribute to the development of maladaptive eating patterns and increase vulnerability to eating-related disorders [1,2].

Contemporary standards regarding body appearance, largely shaped by social media, represent an additional risk factor for the development of EDs among young adults. Constant exposure to idealized images of thin or muscular bodies may contribute to body dissatisfaction, frequent social comparison, and attempts to modify body weight through restrictive eating behaviors [3,4]. It is estimated that EDs affect up to approximately 4% of adolescents and young adults worldwide [5]. Epidemiological data indicate that EDs are diagnosed more frequently among women; however, the actual prevalence among men may be underestimated due to their lower likelihood of seeking professional help, which may result from feelings of shame and the stereotypical perception of ED as predominantly a female-related issue [6].

Regular physical activity is widely recognized as one of the most effective lifestyle strategies for maintaining healthy body weight and improving both physical and psychological health. Current evidence indicates that regular exercise contributes not only to favorable body composition and metabolic health but also to enhanced self-esteem, reduced symptoms of anxiety and depression, and greater body appreciation. Consequently, physical activity is frequently recommended as a sustainable alternative to restrictive dieting for body weight management [7]. However, the relationship between physical activity and eating behaviors appears to be complex. While health-oriented or enjoyment-based exercise is associated with healthier eating habits and better psychological well-being, physical activity driven primarily by appearance- or weight-related motives may coexist with excessive dietary restriction, compulsive exercise, and an increased risk of disordered eating [8]. Therefore, the motivations underlying body weight management strategies, including both restrictive dieting and physical activity, should be considered together when investigating factors associated with ED risk among young adults [9].

University students may demonstrate an increased risk of developing ED compared with individuals of a similar age who do not pursue higher education [10]. A study conducted in 2018 at the University of the Balearic Islands among more than 2000 students aged 18–30 years showed that the risk of developing EDs was 6.1%, with higher values observed among women [1]. Similar trends were observed in studies conducted in Norway, where an increase in the prevalence of EDs was reported between 2018 and 2022 among both female students (from 3.5% to 4.5%) and male students (from 0.4% to 0.6%) [11]. This increase may be partially associated with the consequences of the COVID-19 pandemic, which affected the psychological and social functioning of young individuals [11]. Previous studies indicate that the risk of EDs among university students ranges from approximately 4.5% to 14.3%, highlighting the importance of this issue in the context of public health [12]. In Poland, data regarding the prevalence of EDs among university students remain limited. A study conducted in 2022 among students from the Silesian region using the EAT-26 questionnaire demonstrated that nutrition students did not differ significantly from students in other fields of study in terms of ED risk. At the same time, nearly half of participants in both groups met at least one criterion indicating the presence of abnormal eating behaviors [2].

Restrictive dieting refers to intentional limitation of food intake, food groups, or dietary patterns, often undertaken with the aim of controlling body weight, changing appearance, improving perceived health, or achieving specific dietary goals. Such practices may vary substantially in their nature and motivation, ranging from medically justified dietary modifications (e.g., elimination of specific foods due to diagnosed intolerances or allergies) and ethically or personally motivated dietary choices (e.g., vegetarian or vegan diets) to weight-loss-oriented restrictive practices involving excessive caloric limitation or rigid food rules. Importantly, the presence of dietary restriction alone does not indicate pathological eating behavior; rather, the psychological context, motivation, flexibility, and consequences of restriction determine whether such behaviors may be associated with maladaptive eating patterns [13,14]. In the context of ED vulnerability, particular attention has been given to restrictive practices characterized by rigid dietary rules, excessive concern with body weight or appearance, and inadequate energy intake, such as fasting or very low-calorie diets [15]. Although some dietary patterns may have legitimate health-related applications, inappropriate or excessively restrictive use may contribute to nutritional deficiencies and an unhealthy relationship with food [16,17].

Particular attention should be paid to the increasing popularity of restrictive and elimination diets among young adults. Although some dietary restrictions may have justified applications in specific medical conditions, their inappropriate implementation without professional guidance may contribute to nutritional deficiencies, rigid eating patterns, and an unhealthy relationship with food [16,17,18]. Restrictive practices, including severe caloric limitation, fasting, or unnecessary elimination of specific food groups, are frequently adopted for weight control or perceived health benefits; however, their psychological consequences may differ depending on the motivations underlying these behaviors [17,18,19,20,21].

The motivations underlying the adoption of restrictive eating behaviors are multifactorial and include individual, emotional, and sociocultural factors [22]. One of the most frequently reported reasons is the desire to achieve a specific body shape. Women are more likely to engage in restrictive eating behaviors in order to lose weight due to dissatisfaction with their appearance, whereas men more commonly strive to achieve a muscular physique while maintaining a low level of body fat [3,5]. Social media play an important role in shaping these beliefs by promoting often unrealistic standards of appearance. Trends such as “Thinspiration”, which involve sharing content promoting extremely thin body ideals, may increase body dissatisfaction and encourage the adoption of restrictive eating behaviors [4,23]. A similar risk may be associated with the popular “Clean Eating” movement, which, through categorizing foods as “healthy” or “unhealthy”, may contribute to excessive dietary restriction, particularly among women [24].

A key mechanism explaining the influence of sociocultural appearance standards is appearance ideal internalization, defined as the extent to which individuals adopt socially promoted body ideals as personally meaningful standards for evaluating their own appearance [25,26]. Higher levels of appearance-ideal internalization have been consistently associated with body dissatisfaction, dietary restraint, and symptoms of disordered eating, particularly among young adults exposed to appearance-focused online content [25,26,27,28]. Social media platforms may intensify this process by providing continuous opportunities for comparison with idealized body representations and by normalizing restrictive eating practices as strategies for achieving desirable appearance outcomes. Consequently, appearance internalization may represent an important pathway through which sociocultural pressures translate into restrictive dieting motivations and increased vulnerability to eating-related disturbances.

In addition to the influence of social environment, individual psychological characteristics also play an important role. Individuals characterized by low self-esteem, high levels of perfectionism, and a strong need for control are more likely to engage in restrictive eating behaviors [3,12]. Perfectionism may contribute to rigid adherence to specific dietary rules and the establishment of unrealistic goals regarding body appearance and weight [29]. Moreover, difficulties in emotional regulation may affect eating patterns, as both food restriction and excessive food intake may serve as coping strategies for dealing with stress and emotional distress [30]. Among university students, an additional factor influencing eating behaviors may be stress related to academic demands, which can contribute to meal skipping or reduced food intake [31].

The relationship between restrictive diets and ED has been explained by several theoretical models. One of the most influential is the “restrained eating” theory developed by Polivy and Herman, which suggests that individuals engaging in dietary restriction may experience a loss of control and overeat when previously established dietary rules are violated. This mechanism, referred to as the “what the hell” effect, describes a situation in which a dietary lapse results in the complete abandonment of control and subsequent excessive food consumption [32]. Another important factor is all-or-nothing thinking, in which a single deviation from dietary goals is interpreted as a complete failure [33]. An alternative perspective is provided by Lowe’s Three-Factor Model of Dieting, which suggests that not all forms of dietary restriction lead to ED, and that the key factor is the nature of restriction itself—particularly the distinction between flexible control of food intake and rigid dietary restraint [34]. Long-term dietary restriction may contribute to the restriction–compensation–relapse cycle, disrupt mechanisms regulating hunger and satiety, and increase the risk of developing ED, including bulimia nervosa and binge eating disorder [32,33,34].

Current evidence suggests that restrictive dieting among young adults should be understood as the result of interactions between sociocultural influences, psychological characteristics, and individual motivations. The internalization of socially promoted appearance ideals, particularly those disseminated through social media, may contribute to body dissatisfaction and increased pressure to modify body shape through dietary restriction [3,4,11]. These appearance-related concerns may subsequently influence specific motivations for restrictive dieting, such as weight control, achieving an ideal body shape, or improving self-perceived attractiveness. Importantly, the motivation underlying dietary restriction may determine whether such behaviors represent flexible health-oriented choices or rigid cognitive restraint associated with increased vulnerability to disordered eating [24,34]. Therefore, examining motivational pathways linking restrictive dieting with eating-related behaviors may provide a more comprehensive understanding of ED risk among young adults.

Despite growing interest in the field of ED, factors motivating university students to adopt restrictive eating practices and their relationship with the risk of ED development remain insufficiently understood. Investigating these associations in the population of Polish university students appears particularly important, as epidemiological data regarding this group remain limited. Therefore, the study aimed to analyze the relationship between restrictive dietary practices, eating behaviors, and indicators of vulnerability to disordered eating among university students in Poland. Specifically, the study evaluated the frequency of restrictive diet use, identified the main motivational factors associated with restrictive dieting, and examined their relationships with cognitive restraint as a behavioral indicator of increased vulnerability to disordered eating.

## 2. Materials and Methods

### 2.1. Study Design and Participants

A cross-sectional observational study was conducted between January and May 2025 among university students enrolled at higher education institutions across Poland. The study aimed to assess the prevalence of restrictive dieting, identify the principal motivational factors associated with restrictive dietary practices, and examine their relationship with eating behaviors indicative of increased vulnerability to ED.

Participants were recruited using a convenience sampling strategy through university social media groups, student organizations, and academic online communities. Participation was voluntary and anonymous. Prior to participation, all respondents received information regarding the purpose of the study, confidentiality of collected data, and their right to withdraw at any stage before questionnaire submission.

Eligibility criteria included the following: (1) age between 19 and 34 years; (2) current student status at a Polish university; (3) provision of informed consent; and (4) completion of the questionnaire. Exclusion criteria comprised duplicate submissions, incomplete questionnaires (>10% missing responses), implausible response patterns, and questionnaires completed in less than three minutes.

Initially, 447 questionnaires were collected. Following data verification, 27 questionnaires were excluded because of incompleteness or failure to meet eligibility criteria. Consequently, 420 questionnaires were included in the final statistical analyses. The study population consisted of 285 women (67.9%) and 135 men (32.1%). The characteristics of the students by field of study are presented below (Table 1).

The study was conducted using an anonymous online survey administered through the Google Forms platform. The questionnaire was distributed via social media platforms. To improve data quality and reduce the risk of automated or duplicate submissions, the survey was protected using CAPTCHA verification. Participation was voluntary, and respondents were informed about the purpose of the study and the anonymous nature of data collection.

### 2.2. Sample Size Calculation

The minimum required sample size was estimated using Cochran’s formula for cross-sectional studies:

$$n=\frac{Z^{2}p(1-p)}{d^{2}}$$

where Z = 1.96 (95% confidence level), *p* = 0.50, and d = 0.05.

The required minimum sample size was 385 participants. The final analytical sample comprised 420 respondents, exceeding the minimum required sample by 35 participants.

A post hoc power analysis indicated that the achieved sample size provided 91.8% statistical power to detect small-to-moderate associations (Cohen’s w = 0.20) at α = 0.05.

### 2.3. Ethics

The study was conducted according to the principles of the Declaration of Helsinki. Participation was anonymous and voluntary. No personal identifiers, IP addresses or e-mail addresses were collected. Completion of the questionnaire was considered equivalent to informed consent.

### 2.4. Research Instruments

The Restrictive Diet Motivation Questionnaire (RDMQ) was developed specifically for the purposes of the present study to assess the principal motivational factors underlying restrictive dieting among university students. The development process was theory-driven and based on an extensive review of the literature concerning ED, restrictive dieting, body image, health-related behavior, and sociocultural determinants of dietary restriction. The initial conceptual framework identified five motivational domains consistently reported in previous studies: (1) aesthetic motivation, (2) social motivation, (3) eating control and self-discipline, (4) health-related motivation, and (5) personal beliefs regarding restrictive dieting. Twelve questionnaire items were subsequently constructed to represent these domains using simple and unambiguous language suitable for young adults. Each item was assessed using a five-point Likert scale ranging from 1 (“strongly disagree”) to 5 (“strongly agree”), with higher scores indicating stronger endorsement of a given motivational construct.

The third section included the validated Three-Factor Eating Questionnaire-13 (TFEQ-13), which assesses three dimensions of eating behavior: Cognitive Restraint (CR), Uncontrolled Eating (UE), and Emotional Eating (EE). The instrument has demonstrated satisfactory psychometric properties in previous validation studies and has been extensively applied in studies on eating behaviors among young adults. In the present sample, the TFEQ-13 demonstrated good internal consistency (Cronbach’s α = 0.82), confirming its reliability for use in the current study. The Polish version of the TFEQ-13 has previously demonstrated satisfactory validity and reliability, supporting its use in the assessment of eating behaviors among Polish adults [35].

The Cognitive Restraint (CR) subscale of the TFEQ-13 was selected as the primary outcome because cognitive restraint represents one of the core behavioral dimensions of intentional food restriction. Although the CR subscale is not a diagnostic instrument for ED, higher levels of cognitive restraint have consistently been associated with restrictive eating behaviors and increased vulnerability to disordered eating in non-clinical populations. Accordingly, in the present study, cognitive restraint was interpreted as an indicator of increased ED risk rather than as evidence of a clinical diagnosis.

### 2.5. Psychometric Evaluation of the Author Questionnaire

Before administration, the preliminary version of the questionnaire was independently reviewed by five experts (two clinical psychologists specializing in ED, one psychiatrist, one clinical dietitian, and one public health researcher). The experts evaluated the relevance, clarity, and comprehensiveness of each item using a four-point scale. The Scale Content Validity Index (S-CVI/Ave) was 0.95, indicating excellent content validity. Minor linguistic revisions were introduced following expert recommendations. A pilot study was conducted among 32 university students to assess item clarity and questionnaire comprehensibility. Participants reported no major difficulties in completing the questionnaire. Minor wording modifications were introduced to improve readability before the main survey commenced.

The psychometric properties of the RDMQ were evaluated. Internal consistency analysis demonstrated satisfactory reliability for both the overall questionnaire and the individual motivational domains. The overall scale achieved Cronbach’s α = 0.89 and McDonald’s ω = 0.90, indicating high internal consistency. Cronbach’s α coefficients ranged from 0.79 to 0.88 across the five domains, suggesting satisfactory reliability for each motivational construct. Cronbach’s alpha coefficients for individual domains are presented in Table 2.

Sampling adequacy was confirmed by a Kaiser–Meyer–Olkin index of 0.87, while Bartlett’s test of sphericity was statistically significant (χ^2^ = 1689.4, *df* = 66, *p* < 0.001), confirming the suitability of the data for factor analysis. Principal Axis Factoring with Varimax rotation identified a five-factor solution explaining 71.4% of the total variance. Prior to hypothesis testing, the psychometric properties of the Restrictive Diet Motivation Questionnaire (RDMQ) were evaluated. Internal consistency was assessed using Cronbach’s α and McDonald’s ω coefficients. Construct validity was examined using Exploratory Factor Analysis (EFA) based on Principal Axis Factoring with Varimax rotation. Sampling adequacy was evaluated using the Kaiser–Meyer–Olkin (KMO) statistic and Bartlett’s Test of Sphericity. Factor loadings ≥0.40 were considered acceptable.

### 2.6. Data Quality Control

Data quality procedures included duplicate screening, missing-value analysis, and logical consistency checks.

Questionnaires containing more than 10% missing responses were excluded. Since the remaining dataset contained less than 2% missing values, complete-case analysis was performed.

### 2.7. Statistical Analysis

Statistical analyses were performed using STATISTICA 13.0 (TIBCO Software Inc., Palo Alto, CA, USA). Continuous variables were summarized as means and standard deviations, whereas categorical variables were presented as frequencies and percentages. Normality was evaluated using the Shapiro–Wilk test. Differences between categorical variables were assessed using Pearson’s χ^2^ test or Fisher’s exact test, when appropriate. Effect sizes for χ^2^ analyses were quantified using Cramér’s V, interpreted according to Cohen’s criteria:

- 0.10—small effect;
- 0.30—moderate effect;
- 0.50—large effect.

Associations between ordinal variables were examined using Goodman and Kruskal’s Gamma coefficient (γ) together with 95% confidence intervals. Group comparisons involving continuous non-normally distributed variables were performed using the Mann–Whitney U test or Kruskal–Wallis ANOVA followed by Dunn’s post hoc comparisons. To identify independent predictors of elevated Cognitive Restraint, an ordinal logistic regression model was constructed. Independent variables entered into the model included sex, age, field of study, history of restrictive dieting, aesthetic motivation, social motivation, health motivation, eating control, and personal beliefs regarding restrictive dieting. Model fit was evaluated using the Likelihood Ratio Test, Nagelkerke’s pseudo-R^2^, and the proportional odds assumption was verified using the Test of Parallel Lines. Regression coefficients were reported as adjusted Odds Ratios (aOR) with corresponding 95% confidence intervals. Because multiple hypothesis tests were performed, Benjamini–Hochberg False Discovery Rate correction was applied. All analyses were conducted using two-sided statistical tests, with *p* < 0.05 considered statistically significant.

## 3. Results

### 3.1. Restrictive Dieting Practices Among the Studied Group of Students

In the studied group, the majority of respondents had not followed restrictive diets either in the past or at the time of the study (n = 255; 61%). A total of 165 participants (39%) reported having followed restrictive diets at some point in their lives. Women declared the use of restrictive diets more frequently than men. Restrictive dieting was reported by 120 women (42.11%) and 45 men (33.33%). Among the analyzed fields of study, the majority of respondents declared that they had not followed restrictive diets. The highest percentage of individuals reporting restrictive dieting was observed among students from Engineering and Computer Science (n = 27; 47.37%), the Humanities and Social Sciences (n = 27; 42.86%), and Medical and Life Sciences (n = 57; 41.30%). The lowest prevalence of restrictive dieting was reported among students from the Arts (n = 18; 28.57%). The unexpectedly higher prevalence of restrictive dieting among Engineering and Computer Science students may reflect subgroup-specific body image pressures, lifestyle characteristics, or sampling-related factors; however, this finding requires further investigation in representative student populations.

### 3.2. Analysis of Factors Influencing Restrictive Dieting

The responses of all participants to the questions included in the original questionnaire were analyzed. The highest number of respondents agreed with statements related to aesthetic factors, such as the desire to have an attractive appearance, the influence of a slim figure on self-esteem, and the need to lose weight in order to look better in clothes, as well as with social factors, including the desire to belong to a group of people who follow healthy eating practices and comparing oneself with individuals on social media. Health-related motivations were the least frequently reported, such as restricting food intake to prevent diseases, and personal beliefs regarding restrictive diets, such as the belief that a restrictive diet is the only way to maintain body weight.

A detailed analysis was conducted among respondents who reported currently following or having previously followed restrictive diets. The questionnaire items were divided into five groups of factors: aesthetic, social, related to eating control and self-discipline, health-related, and personal beliefs about restrictive diets.

**Figure 1:**
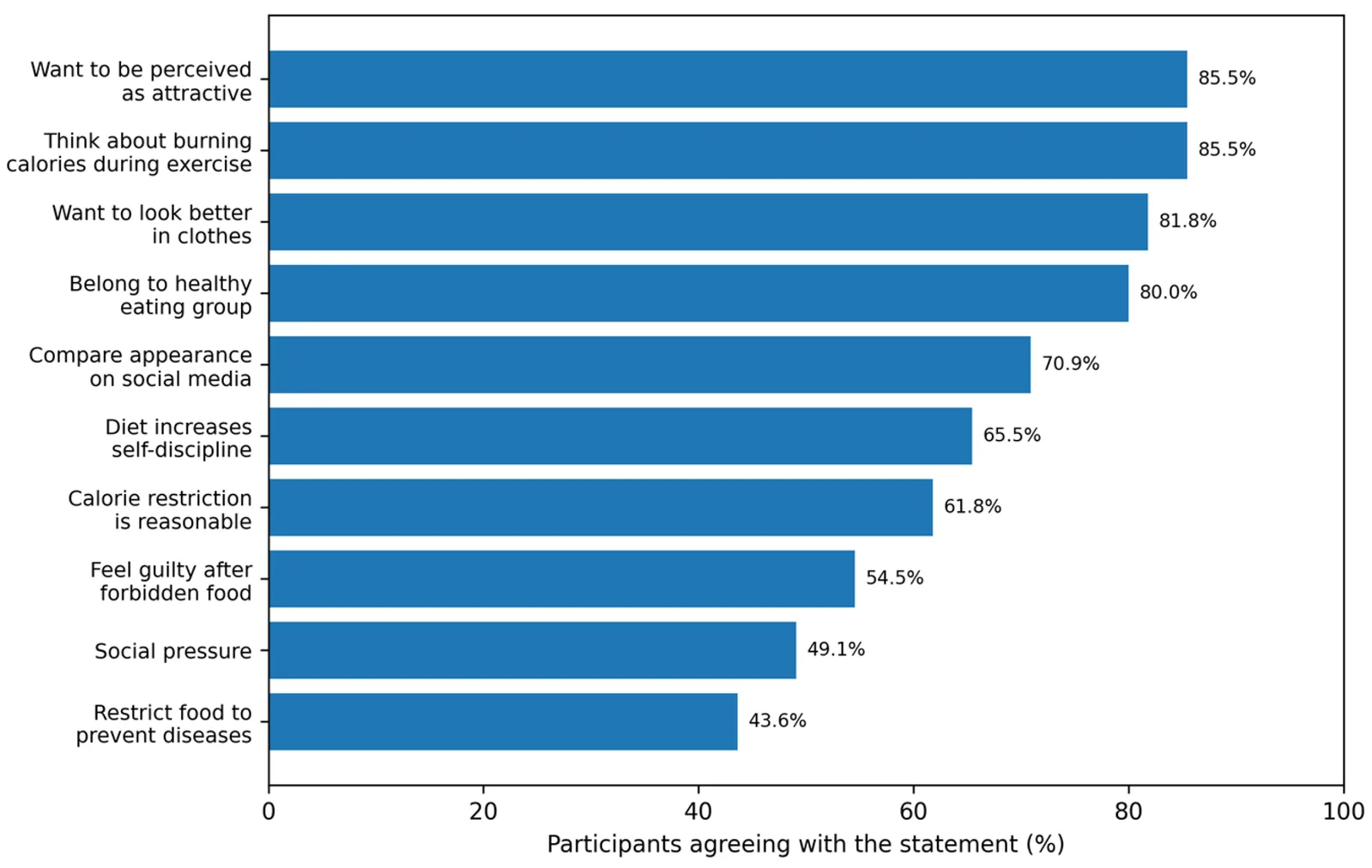
Motivations for restrictive dieting among university students who reported current or previous use of restrictive diets (n = 165). Values represent the percentage of participants who agreed with each statement.

Among individuals who engaged in restrictive eating behaviors, aesthetic motivations were predominant. The vast majority of participants wanted to be perceived as attractive (n = 141; 85.45%) and wanted to lose weight in order to look better in clothes (n = 135; 81.82%). Among social factors, the strongest motivation was the desire to belong to a group of people who care about healthy eating, which was reported by 132 participants (80.00%). Comparing one’s appearance with people on social media was declared by 117 respondents (70.91%). Perceived social pressure was reported by 81 participants (49.09%). The majority of respondents (n = 141; 85.45%) declared that they think about burning calories during exercise. Feelings of guilt after eating something considered forbidden were reported by 90 participants (54.55%). Health-related motivation was the least frequently reported factor—only 72 participants (43.64%) restricted their food intake to prevent diseases, whereas 60 participants (36.36%) disagreed with this statement. More than half of the participants (n = 102; 61.82%) considered calorie restriction to be reasonable. The majority of respondents (n = 108; 65.45%) reported feeling more disciplined due to following a restrictive diet. At the same time, almost half of the participants (n = 78; 47.27%) disagreed that a restrictive diet is the only way to maintain body weight (Figure 1).

### 3.3. Eating Behaviors in the Studied Group of Students

The analysis of the TFEQ-13 questionnaire showed that the studied students most frequently responded to external food-related stimuli—the sight or smell of food encouraged the majority of them to eat. In the domain of cognitive restraint, more than half of the respondents reported consciously controlling their portion sizes. Emotional eating, defined as consuming meals in response to stress, was declared by approximately half of the participants.

Regarding cognitive restraint, both women (n = 201; 70.53%) and men (n = 84; 62.22%) presented a moderate level of restriction. In the domain of uncontrolled eating, a moderate level predominated in both sexes; however, women (n = 84; 49.47%) more frequently achieved a high level of uncontrolled eating compared with men (n = 12; 8.89%). In terms of emotional eating, a moderate level was the most prevalent, although men more frequently than women demonstrated a high level of emotional eating.

A moderate level of cognitive restraint predominated in all fields of study. The highest proportion was observed among students from Economics and Law (n = 78; 78.79%). A high level of restraint was uncommon across all groups; however, it occurred most frequently among students from Medical and Life Sciences (n = 18; 13.04%). Students from Engineering and Computer Science least frequently presented a low level of cognitive restraint (n = 18; 31.58%).

A moderate level of uncontrolled eating predominated in all groups. Students from Medical and Life Sciences differed from the other groups, as they most frequently presented a low level of uncontrolled eating (n = 51; 36.96%) and least frequently a moderate level (n = 72; 52.17%).

A moderate level of emotional eating was predominant across all groups. A low level of emotional eating was most frequently observed among students from Engineering and Computer Science (n = 24; 42.11%), whereas the highest level was reported among students from Humanities and Social Sciences (n = 9; 14.29%) and Medical and Life Sciences (n = 15; 10.87%).

Individuals who followed restrictive diets more frequently demonstrated moderate and high levels of cognitive restraint compared with those who did not follow restrictive diets. Among participants using restrictive diets, a high level of cognitive restraint was observed in 24 respondents (14.55%), whereas in the group not following restrictive diets, it was reported by 9 respondents (3.53%). A statistically significant association was found between the analyzed variables (χ^2^ = 20.35; *p* < 0.001).

### 3.4. Analysis of Factors Motivating Restrictive Dieting and the Risk of Developing Eating Disorders

**Figure 2:**
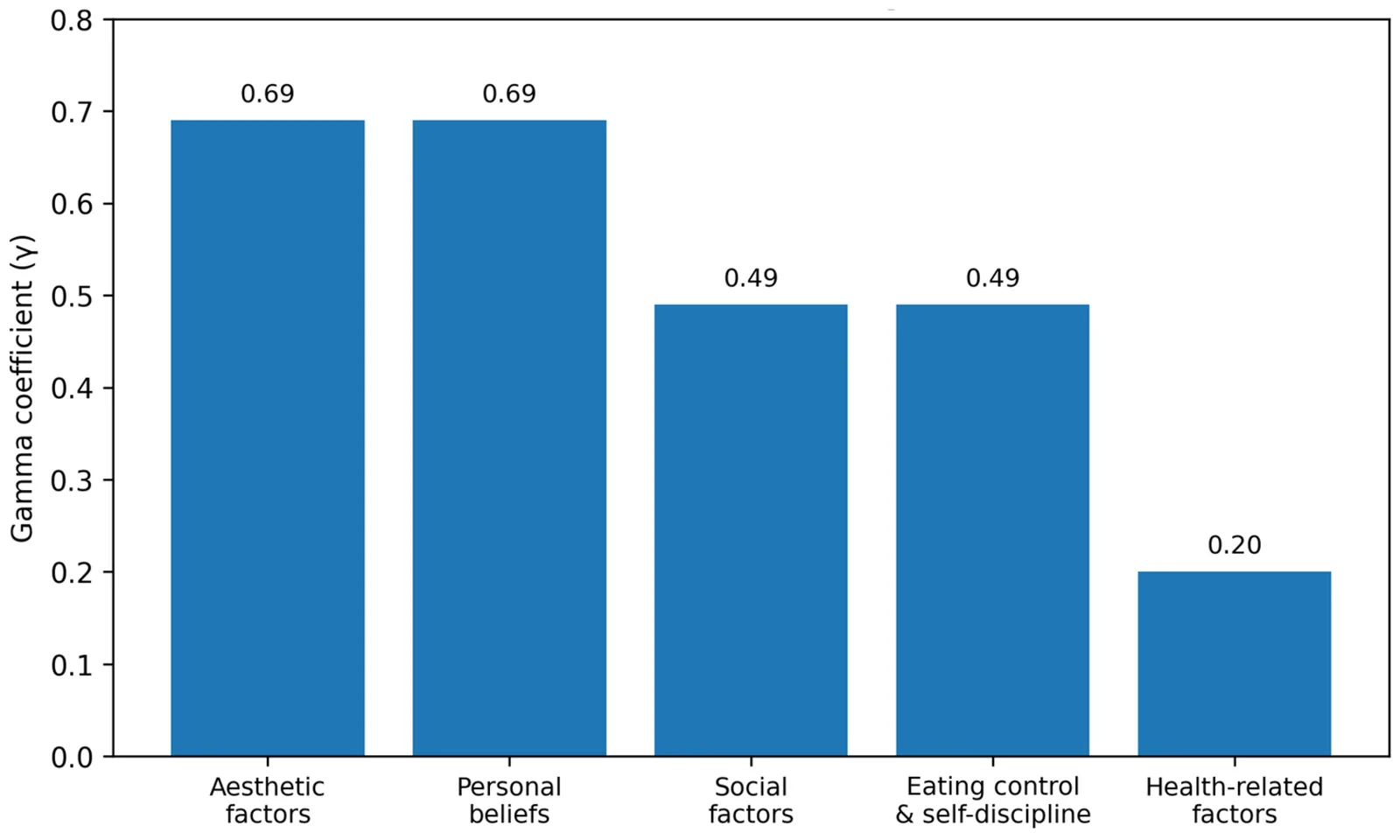
Strength of associations between motivational domains for restrictive dieting and eating behavior patterns associated with the development of restrictive eating tendencies. The bars represent Gamma (γ) correlation coefficients between each motivational domain and cognitive restraint (TFEQ-13), used as an indicator of ED risk.

Five groups of factors motivating individuals to follow restrictive diets were compared with the risk of developing ED. The risk of ED was assessed using the R—Cognitive Restraint subscale of the standardized TFEQ-13 questionnaire (Table 3; Figure 2).

The analysis of the relationship between aesthetic factors and the risk of developing ED revealed a statistically significant association (χ^2^ = 21.41; *p* < 0.001). The Gamma coefficient value was 0.69, indicating a strong correlation. Among individuals with high aesthetic motivations, 195 participants (73.86%) demonstrated a moderate level of cognitive restraint, while 33 (12.50%) demonstrated a high level, indicating an increased risk of developing ED. Among individuals with low aesthetic motivations, none of the participants achieved a high level of cognitive restraint.

The analysis of the relationship between social factors and the risk of developing ED showed a statistically significant association (χ^2^ = 14.13; *p* < 0.007). The Gamma coefficient value was 0.49, indicating a moderate correlation. Among individuals with high social motivations, 156 participants (75.36%) demonstrated a moderate level of cognitive restraint, and 24 participants (11.59%) demonstrated a high level.

The analysis of the relationship between factors related to eating control and self-discipline and the risk of developing ED showed a statistically significant association (χ^2^ = 13.28; *p* < 0.010). The Gamma coefficient value was 0.49, indicating a moderate correlation. Among individuals with high motivation related to eating control and self-discipline, 123 participants (82.00%) demonstrated a moderate level of cognitive restraint, while 15 participants (10.00%) demonstrated a high level.

The analysis of the relationship between health-related motivations and the risk of developing ED was statistically significant (χ^2^ = 19.38; *p* < 0.001). The Gamma coefficient value (γ) was 0.20, indicating a weak correlation. Among individuals with low levels of health-related motivations, 60 participants (76.92%) demonstrated a low level of cognitive restraint, whereas among individuals with high levels of health-related motivations, moderate cognitive restraint predominated (n = 120; 78.43%).

A statistically significant association was found between personal beliefs and attitudes regarding restrictive diets and eating behavior patterns associated with restrictive eating tendencies (χ^2^ = 28.11; *p* < 0.001). The obtained Gamma coefficient value (γ) was 0.69, demonstrating a strong correlation. As the intensity of personal beliefs regarding restrictive diets increased, the eating behavior patterns associated with restrictive eating tendencies also increased.

An ordinal logistic regression model was constructed to identify independent predictors of higher Cognitive Restraint scores. The overall model was statistically significant (Likelihood Ratio χ^2^ = 86.43; *df* = 9; *p* < 0.001) and demonstrated satisfactory fit (Nagelkerke R^2^ = 0.36). The proportional odds assumption was not violated (Test of Parallel Lines: χ^2^ = 11.28; *p* = 0.42). Aesthetic motivation remained the strongest independent predictor of higher cognitive restraint (aOR = 2.84; 95% CI: 1.96–4.11; *p* < 0.001). Personal beliefs regarding restrictive dieting were also independently associated with higher cognitive restraint (aOR = 2.17; 95% CI: 1.49–3.16; *p* < 0.001). Previous restrictive dieting (aOR = 1.94; 95% CI: 1.28–2.96; *p* = 0.002) and female sex (aOR = 1.61; 95% CI: 1.08–2.41; *p* = 0.021) were additional independent predictors. Social motivation showed a weaker but statistically significant association (aOR = 1.38; 95% CI: 1.03–1.86; *p* = 0.034), whereas health-related motivation was not independently associated with cognitive restraint after adjustment (aOR = 1.12; 95% CI: 0.83–1.51; *p* = 0.462)—Table 4.

## 4. Discussion

The multivariable analysis strengthened the findings obtained in the univariate analyses. After adjustment for demographic characteristics and dieting history, aesthetic motivation remained the strongest predictor of elevated cognitive restraint. This suggests that appearance-related motives represent an independent psychological correlate of restrictive eating behavior rather than merely reflecting differences in sex or previous dieting experience. Personal beliefs regarding restrictive dieting also remained independently associated with cognitive restraint, highlighting the importance of dieting-related cognitions in ED vulnerability.

Nowadays, the use of restrictive diets has become a widespread phenomenon, particularly among young people who are entering adulthood and facing various everyday challenges for the first time. In the study by Souto et al., conducted among 251 Brazilian university students, 96 participants (38.30%) reported having followed restrictive diets [36]. Therefore, these results were nearly identical to those obtained in the present study. It is worth noting the differences in adopting dietary restrictions according to participants’ sex. In the present study, women (n = 120; 42.11%) more frequently reported following restrictive diets than men (n = 45; 33.33%). This finding is consistent with observations reported in the scientific literature. Similar results were obtained by Kriaučionienė et al., who showed that 259 female students (52.50%) and 51 male students (23.60%) had attempted to reduce their body weight at some point in the past. The study included first-year university students [37]. The more frequent use of restrictive diets among women may result from greater appearance-related expectations toward women, which are deeply rooted in cultural norms. This sex difference may be partly explained by greater exposure of women to appearance-related pressures and stronger internalization of thin-ideal standards. Social media platforms may amplify these pressures by promoting highly curated body images and encouraging frequent appearance comparison [26,27,28]. Such processes may contribute to body dissatisfaction, which has been identified as an important mediator between sociocultural influences and restrictive eating behaviors.

In the present study, aesthetic factors predominated among individuals who decided to follow restrictive diets, including the desire to lose weight in order to look better in clothes (n = 135; 81.82%) and the desire to be perceived as an attractive person (n = 141; 85.45%). Similar observations have been reported by other researchers. A study conducted among 308 university students from the United Arab Emirates aged 18–25 years showed that 80.9% of respondents were dissatisfied with their bodies [38]. Another study conducted in China demonstrated a statistically significant association between body dissatisfaction and the use of restrictive diets [39]. This indicates that individuals with a more negative perception of their appearance are more likely to engage in dietary restriction. However, body dissatisfaction alone does not fully explain restrictive dieting. The psychological mechanism may involve appearance ideal internalization, whereby individuals adopt culturally promoted body standards as personal goals. When these standards become central to self-evaluation, restrictive dieting may be perceived as an effective strategy for achieving social acceptance, improving self-worth, or reducing dissatisfaction with one’s appearance. Research by Vartanian et al. showed that individuals following restrictive diets were more motivated to lose weight for appearance-related reasons than those who did not engage in restrictive dieting [40]. In the present study, health-related motivation was the least frequently reported factor and was observed only among 72 participants who declared restrictive dieting (43.64%). Interestingly, Vartanian et al. found that individuals who did not follow restrictive diets were more likely to report weight-loss motivation related to health reasons [41].

The study based on the TFEQ-13 questionnaire demonstrated that women more frequently exhibited a high level of cognitive restraint (n = 27; 9.47%) and a high level of uncontrolled eating (n = 84; 29.47%). In contrast, men more frequently demonstrated a high level of emotional eating (n = 27; 20.00%). A 2021 study conducted among Chinese university students revealed that women achieved higher scores than men in all three TFEQ-13 subscales, including cognitive restraint, emotional eating, and uncontrolled eating [33]. A Polish study also provided a different perspective on the relationship between TFEQ-13 results and participants’ sex. Kowalkowska and Poínhos, using the TFEQ-13 questionnaire, showed that cognitive restraint and emotional eating were higher among women, whereas men more frequently experienced loss of control over eating [42]. Across the three studies compared above, consistency was observed regarding higher levels of cognitive restraint among women, while differences were found in terms of emotional eating and uncontrolled eating.

In the present study, the highest percentage of individuals with a high level of cognitive restraint according to the TFEQ-13 was observed among students from Medical and Life Sciences (n = 18; 13.04%). A study conducted in 2024 among students of the Medical University of Silesia in Katowice, the Jerzy Kukuczka Academy of Physical Education in Katowice, the University of Silesia in Katowice, and the University of Economics in Katowice confirmed that students from health-related fields achieved higher TFEQ-13 cognitive restraint scores compared with students from other fields of study [43]. Students of dietetics and physical education were classified as students of health-related disciplines, whereas students of management and computer science were classified as students of non-health-related disciplines. The results of the present study did not confirm the findings of the previously analyzed study among Silesian students, in which uncontrolled eating and emotional eating measured by TFEQ-13 were more frequently observed among students from non-health-related fields, reaching 15.20% and 73.5%, respectively [43]. In the present study, students from Medical and Life Sciences demonstrated both high levels of cognitive restraint (n = 15; 10.87%) and low levels (n = 51; 36.96%), which distinguished this group from other fields of study. Regarding emotional eating assessed by TFEQ-13, a moderate level was the most frequently observed category in all groups of academic disciplines.

The present study indicated that individuals following restrictive diets more frequently demonstrated a high level of cognitive restraint measured by TFEQ-13 (n = 24; 14.55%). A similar tendency was observed in a study conducted among Polish women aged 19–30 years, in which participants following vegetarian and Low FODMAP diets achieved higher mean scores for cognitive restraint according to TFEQ-13 compared with individuals following a traditional diet [44]. In the present study, the majority of respondents reported that they wanted to be perceived as attractive or that they wanted to lose weight in order to look better in clothes. Furthermore, a strong correlation (γ = 0.69) was found between aesthetic motivations and the risk of developing ED, expressed by a high level of cognitive restraint according to TFEQ-13. Different results were obtained by Tey et al., who found no significant differences in cognitive restraint among university students with appropriate and inappropriate body image perceptions [45]. These discrepancies may result from methodological differences, as the present study analyzed aesthetic motivations leading to restrictive dieting, whereas the authors of the cited study compared only body image perception. Findings closer to those of the present study were reported by Yong et al., who found a high level of cognitive restraint among 489 students dissatisfied with their appearance (61.00%) and among 198 students satisfied with their appearance (39.7%) [41]. The conducted analyses suggest that greater importance attributed to physical appearance and attractiveness may contribute to the adoption of restrictive eating behaviors and simultaneously increase the risk of developing ED among university students.

The present findings may also be interpreted in the broader context of physical activity, which is considered one of the most important components of a healthy lifestyle and a recommended strategy for sustainable body weight management. Numerous studies have demonstrated that regular physical activity is associated with better psychological well-being, improved body image, and healthier eating behaviors, particularly when participation is motivated by health, enjoyment, or personal well-being rather than appearance-related goals. In contrast, individuals who exercise primarily to modify their physical appearance or lose weight may be more likely to engage in maladaptive behaviors, including restrictive dieting and compulsive exercise, both of which have been linked to increased eating behavior patterns associated with restrictive eating tendencies. Although physical activity was not assessed in the present study, the findings may have implications for broader weight-management behaviors. Previous research suggests that appearance-oriented approaches to physical activity may coexist with restrictive dieting and other maladaptive behaviors. Therefore, future studies should consider both dietary and exercise-related motivations when investigating ED vulnerability among young adults [41,43,44,45].

A similar relationship was observed regarding personal beliefs and attitudes toward restrictive diets. In the present study, a strong correlation was found between the intensity of these beliefs and the risk of developing ED (γ = 0.69). This finding is partially supported by the study of Mata et al., who indicated that attitudes toward dieting, eating patterns, and body weight play an important role in shaping health-related behaviors [46]. Moreover, the authors emphasized that attitudes influence dietary intentions and motivations. The obtained results suggest that stronger beliefs regarding the necessity and effectiveness of restrictive diets may coexist with a higher risk of behaviors characteristic of ED.

## 5. Strengths, Limitations, and Future Perspectives

This study has several strengths. First, it included a relatively large sample of university students representing a broad range of academic disciplines, allowing comparisons across different fields of study. Second, the use of the validated Three-Factor Eating Questionnaire-13 (TFEQ-13) provided a standardized and psychometrically sound assessment of eating behaviors, facilitating comparisons with previous studies. Third, the study introduced the Restrictive Diet Motivation Questionnaire (RDMQ), a theory-driven instrument developed to assess motivational factors underlying restrictive dieting. The questionnaire demonstrated satisfactory preliminary psychometric properties, including good internal consistency and a stable five-factor structure, supporting its application in the present study. Furthermore, by combining the assessment of eating behaviors with an evaluation of motivations for restrictive dieting, the study provides a more comprehensive understanding of psychological factors associated with restrictive eating practices and vulnerability to disordered eating among university students.

Several limitations should be acknowledged. First, the cross-sectional design prevents conclusions regarding causal relationships between restrictive dieting motivations and eating behaviors. Longitudinal studies are required to determine whether specific motivational patterns precede the development of more severe disordered eating symptoms over time. Second, all data were collected using self-report questionnaires, which may be affected by recall bias and social desirability bias. Third, participants were recruited using a non-probabilistic convenience sampling strategy through university social media groups and online student communities. Therefore, selection bias cannot be excluded, and the sample may not fully represent the broader population of Polish university students.

Additionally, several potentially important variables were not assessed. The absence of anthropometric measurements, particularly body mass index (BMI), limits the ability to determine whether motivations for restrictive dieting differ according to weight status. Furthermore, psychological factors such as anxiety, depression, self-esteem, perfectionism, and body dissatisfaction were not evaluated, although these variables may simultaneously influence dieting motivations, body image concerns, and eating behaviors. Other potentially relevant factors, including socioeconomic status, physical activity level, psychiatric history, alcohol consumption, use of weight-loss medications, and daily exposure to social media, were also not assessed and may represent important sources of residual confounding.

Moreover, women constituted more than two-thirds of the study population, which may have influenced the observed sex-related differences despite adjustment for sex in the multivariable analyses. It should also be emphasized that the Cognitive Restraint subscale of the TFEQ-13 was not used as a diagnostic measure of ED. Rather, it was interpreted as a behavioral indicator of intentional dietary restriction and increased vulnerability to disordered eating. Therefore, the findings should be interpreted as reflecting elevated eating disorder risk indicators rather than clinically diagnosed ED. Finally, although the RDMQ demonstrated satisfactory preliminary psychometric properties, further validation studies in independent populations, including confirmatory factor analysis, test–retest reliability, and measurement invariance assessment, are warranted.

The findings of this study may have important implications for prevention and health promotion among university students. Universities could benefit from implementing educational programs focused on evidence-based nutrition knowledge, critical evaluation of diet-related information, media literacy, and promotion of a positive body image. Nutrition professionals and psychologists should consider not only the presence of restrictive eating behaviors but also the motivations underlying these practices, particularly appearance-related motives and rigid beliefs regarding dieting. Early identification of students with high cognitive restraint combined with strong appearance concerns may support preventive interventions aimed at reducing the risk of unhealthy dietary restriction and disordered eating patterns.

Future research should adopt longitudinal designs to clarify whether specific motivational profiles predict the development of clinically diagnosed ED or persistent disordered eating patterns over time. Future investigations should also incorporate objective anthropometric measurements and comprehensive psychological assessments, including anxiety, depression, perfectionism, self-esteem, and body dissatisfaction, as well as evaluate physical activity levels, social media exposure, and socioeconomic factors to better understand the complex determinants of restrictive dieting behaviors. Multicenter studies involving more diverse and representative populations from different countries would improve external validity and allow cross-cultural comparisons. Additionally, intervention studies evaluating educational and psychological programs focused on healthy body image, media literacy, and evidence-based nutrition could help identify effective strategies for reducing unhealthy restrictive dieting among university students.

## 6. Conclusions

The study findings indicate that women were more likely than men to follow restrictive diets and to achieve higher scores on cognitive restraint as assessed using the TFEQ-13 questionnaire. Aesthetic concerns were the most frequently reported motivation for adopting restrictive diets, and individuals following such diets simultaneously exhibited higher levels of cognitive restraint. Importantly, both appearance-related motivations and personal beliefs regarding restrictive dieting were most strongly associated with an increased risk of developing ED. These findings highlight the need for educational and preventive interventions aimed at promoting evidence-based nutrition knowledge, fostering a healthy body image, and reducing the influence of appearance-related motivations on dietary choices.

## Figures and Tables

**Table 1:** Characteristics of students by field of study.

Field of Study	N	%
Medical and Life Sciences (e.g., medicine, nursing, midwifery, dietetics, biology)	138	32.86%
Economics and Law (e.g., management, law, marketing, finance and accounting)	99	23.57%
Humanities and Social Sciences (e.g., psychology, philology, pedagogy, sociology)	63	15.00%
Arts (e.g., interior architecture, fine arts, musicology, film studies)	63	15.00%
Engineering and Computer Science (e.g., computer science, civil engineering, robotics and automation, electronics)	57	13.57%

**Table 2:** Cronbach’s alpha coefficients for individual domains of the Author Questionnaire.

Domain	Cronbach’s α
Aesthetic	0.88
Social	0.84
Eating control	0.81
Health	0.79
Personal beliefs	0.86

**Table 3:** Summary of associations between restrictive dieting motivations and ED risk.

Motivation Domain	χ^2^	Gamma (γ)	*p*-Value	Strength of Association
Aesthetic	21.41	0.69	<0.001	Strong
Social	14.13	0.49	0.007	Moderate
Eating control/self-discipline	13.28	0.49	0.010	Moderate
Health-related	19.38	0.20	<0.001	Weak
Personal beliefs	28.11	0.69	<0.001	Strong

**Table 4:** Ordinal logistic regression analysis identifying independent predictors of higher cognitive restraint (TFEQ-13).

Variable	aOR	95% CI	*p*
**Female sex**	1.61	1.08–2.41	0.021
**Previous restrictive dieting**	1.94	1.28–2.96	0.002
**Aesthetic motivation**	2.84	1.96–4.11	<0.001
**Social motivation**	1.38	1.03–1.86	0.034
**Health motivation**	1.12	0.83–1.51	0.462
**Eating control**	1.47	1.09–1.98	0.011
**Personal beliefs**	2.17	1.49–3.16	<0.001

## Data Availability

The data presented in this study are available from the corresponding author upon reasonable request. The data are not publicly available due to privacy and ethical restrictions.
